# Ensemble-AHTPpred: A Robust Ensemble Machine Learning Model Integrated With a New Composite Feature for Identifying Antihypertensive Peptides

**DOI:** 10.3389/fgene.2022.883766

**Published:** 2022-04-28

**Authors:** Supatcha Lertampaiporn, Apiradee Hongsthong, Warin Wattanapornprom, Chinae Thammarongtham

**Affiliations:** ^1^ Biochemical Engineering and Systems Biology Research Group, National Center for Genetic Engineering and Biotechnology, National Science and Technology Development Agency at King Mongkut’s University of Technology Thonburi, Bangkok, Thailand; ^2^ Applied Computer Science Program, Department of Mathematics, Faculty of Science, King Mongkut’s University of Technology Thonburi, Bangkok, Thailand

**Keywords:** antihypertensive, prediction, classification, ACE inhibitor, ACE inhibitory peptide, ensemble machine learning

## Abstract

Hypertension or elevated blood pressure is a serious medical condition that significantly increases the risks of cardiovascular disease, heart disease, diabetes, stroke, kidney disease, and other health problems, that affect people worldwide. Thus, hypertension is one of the major global causes of premature death. Regarding the prevention and treatment of hypertension with no or few side effects, antihypertensive peptides (AHTPs) obtained from natural sources might be useful as nutraceuticals. Therefore, the search for alternative/novel AHTPs in food or natural sources has received much attention, as AHTPs may be functional agents for human health. AHTPs have been observed in diverse organisms, although many of them remain underinvestigated. The identification of peptides with antihypertensive activity in the laboratory is time- and resource-consuming. Alternatively, computational methods based on robust machine learning can identify or screen potential AHTP candidates prior to experimental verification. In this paper, we propose Ensemble-AHTPpred, an ensemble machine learning algorithm composed of a random forest (RF), a support vector machine (SVM), and extreme gradient boosting (XGB), with the aim of integrating diverse heterogeneous algorithms to enhance the robustness of the final predictive model. The selected feature set includes various computed features, such as various physicochemical properties, amino acid compositions (AACs), transitions, n-grams, and secondary structure-related information; these features are able to learn more information in terms of analyzing or explaining the characteristics of the predicted peptide. In addition, the tool is integrated with a newly proposed composite feature (generated based on a logistic regression function) that combines various feature aspects to enable improved AHTP characterization. Our tool, Ensemble-AHTPpred, achieved an overall accuracy above 90% on independent test data. Additionally, the approach was applied to novel experimentally validated AHTPs, obtained from recent studies, which did not overlap with the training and test datasets, and the tool could precisely predict these AHTPs.

## Introduction

Hypertension is a global health issue due to its worldwide incidence and association with increased mortality and morbidity (Mills et al., 2020). Chronic hypertension is a substantial risk factor for heart diseases, stroke, cardiovascular diseases, congestive heart failure, glomerulonephritis, arteriosclerosis, and other diseases (Zhou et al., 2021).

The renin-angiotensin system (RAS) or the renin-angiotensin-aldosterone system (RAAS) is responsible for blood pressure regulation. The RAS regulates blood pressure and cardiac output by controlling the flow of blood through the heart (Wu et al., 2018).

One of the most important enzymes in the RAS system, angiotensin-converting enzyme (ACE), regulates blood pressure and fluid/salt homeostasis (He et al., 2014; Balgir and Sharma 2017). In the RAS, renin transforms angiotensinogen into angiotensin-I (ANG I), and subsequently, ACE transforms the inactive decapeptide angiotensin-I (ANG I) into the vasoconstrictor octapeptide angiotensin-II (ANG II). Excessive ACE activity results in the production of excessive amounts of angiotensin II and, as a result, an increase in blood pressure (i.e., it upregulates blood pressure) (Zhu et al., 2021).

ACE inhibition is a well-established technique for developing pharmaceuticals for the treatment of hypertension. Synthetic ACE inhibitors such as captopril, enalapril, cilazapril, benazepril, and lisinopril are typically used in clinical hypertension treatments (Daskaya-Dikmen, et al., 2017). However, the long-term treatment of hypertension with these drugs is accompanied by severe or mild adverse effects, such as cough, headache, diarrhea, dizziness, fatigue, angioedema, hyperkalemia, hypotension, or, in rare cases, renal impairment (De Leo et al., 2009; Nguyen et al., 2010; Norris and FitzGerald, 2013; Daskaya-Dikmen et al., 2017; Abachi et al., 2019; Festa et al., 2020).

Antihypertensive peptides (AHTPs) are bioactive peptides obtained from natural foods that have the effects/activities of ACE inhibitors against hypertension and are considered safe for consumption, with fewer adverse side effects than synthetic ACE inhibitor drugs or even no side effects. These natural ACE inhibitory bioactive peptides are highly desired for the development of functional foods, nutraceuticals and pharmaceuticals for the prevention and treatment of hypertension (Norris and FitzGerald, 2013; de Castro and Sato, 2015; Kumar et al., 2015; Abachi et al., 2019; Pujiastuti et al., 2019; Jiang et al., 2021; Zaky et al., 2022). Peptides are often multifunctional and may exhibit several health-promoting bioactivities, such as antioxidative, antihypertensive, anti-inflammatory, cytoprotective, and antimicrobial effects (He et al., 2019; Jakubczyk et al., 2020). Emerging evidence indicates that AHTPs may mediate antihypertensive effects by interacting with RAS-related renin, AT-II receptors, arginine–nitric oxide pathway, endothelin system, or Ca^2+^ channels in addition to ACE inhibition (Udenigwe and Mohan, 2014; Aluko 2015). AHTPs have major potential as functional ingredients (dietary compounds) in a daily diet aimed at helping prevent and safely manage hypertension and enhancing human health (Norris and FitzGerald, 2013; Jakubczyk et al., 2020). Therefore, the identification of new, nontoxic bioactive peptides derived from food or natural sources has received significant attention. As a consequence, an increasing number of food-derived antihypertensive peptides have been studied and reported (Martínez-Maqueda et al., 2012; Kumar et al., 2014; Abachi et al., 2019; Lee and Hur 2019; Pujiastuti et al., 2019; Lu et al., 2021). Finding new AHTPs in various organisms is currently a significant research topic. However, large-scale identification through wet laboratory experiments is a costly, time consuming, and labor-intensive approach (Li-Chan 2015; Pujiastuti et al., 2019; Festa et al., 2020). The use of bioinformatics and *in silico* methods for the identification of potential candidate AHTPs for subsequent experimental assays is necessary to shorten the process. The development of efficient computational approaches will facilitate the processes of discovery and screening, allowing potential novel AHTP candidates to be identified in a cost-, resource- and time-effective manner.

A few existing machine learning-based computational approaches are available for predicting AHTPs. mAHTPred is a meta-predictor that employs a two-step feature selection methodology (Manavalan et al., 2019). PAAP is an RF classification model approach based on varied combinations of amino acids, dipeptides, and pseudo amino acid composition descriptors (Win et al., 2018). AHTpin was developed to screen, predict, and design AHTPs by using an SVM-based regression model for tiny peptides and SVM-based classification models for small, medium and large peptides (Kumar et al., 2015). Additionally, an SVM prediction tool was recently built by using convolutional neural network (CNN) deep learning-based encoding features derived from amino acid compositions (AACs) and dipeptide composition features (Rauf et al., 2021).

Although certain tools for AHTP prediction are available, the development of our ensemble method is different from that of the existing approaches in several ways. First, we developed a weighted voting method for integrating the strengths of three independent machine learning models, each of which has high levels of performance in different aspects. Second, a new composite feature called comF2 was developed based on a logistic regression statistical framework. In both the RF and extreme gradient boosting (XGB) feature importance plots, this feature was ranked as the most significant. In addition, a Shapley additive explanations (SHAP) analysis revealed consistent results, showing that comF2 was the top-ranked feature and was capable of explaining large samples in the model; therefore, it could capture characteristics for most of the AHTPs in the training data. Third, our ensemble method outperformed previously developed methods in terms of robustness and accuracy when predicting independent testing datasets, with an enhanced accuracy of 90.4%. Last, the technique could also correctly classify many novel unseen, and experimental AHTPs collected from recent studies.

## Materials and Methods

### Workflow

**FIGURE 1:**
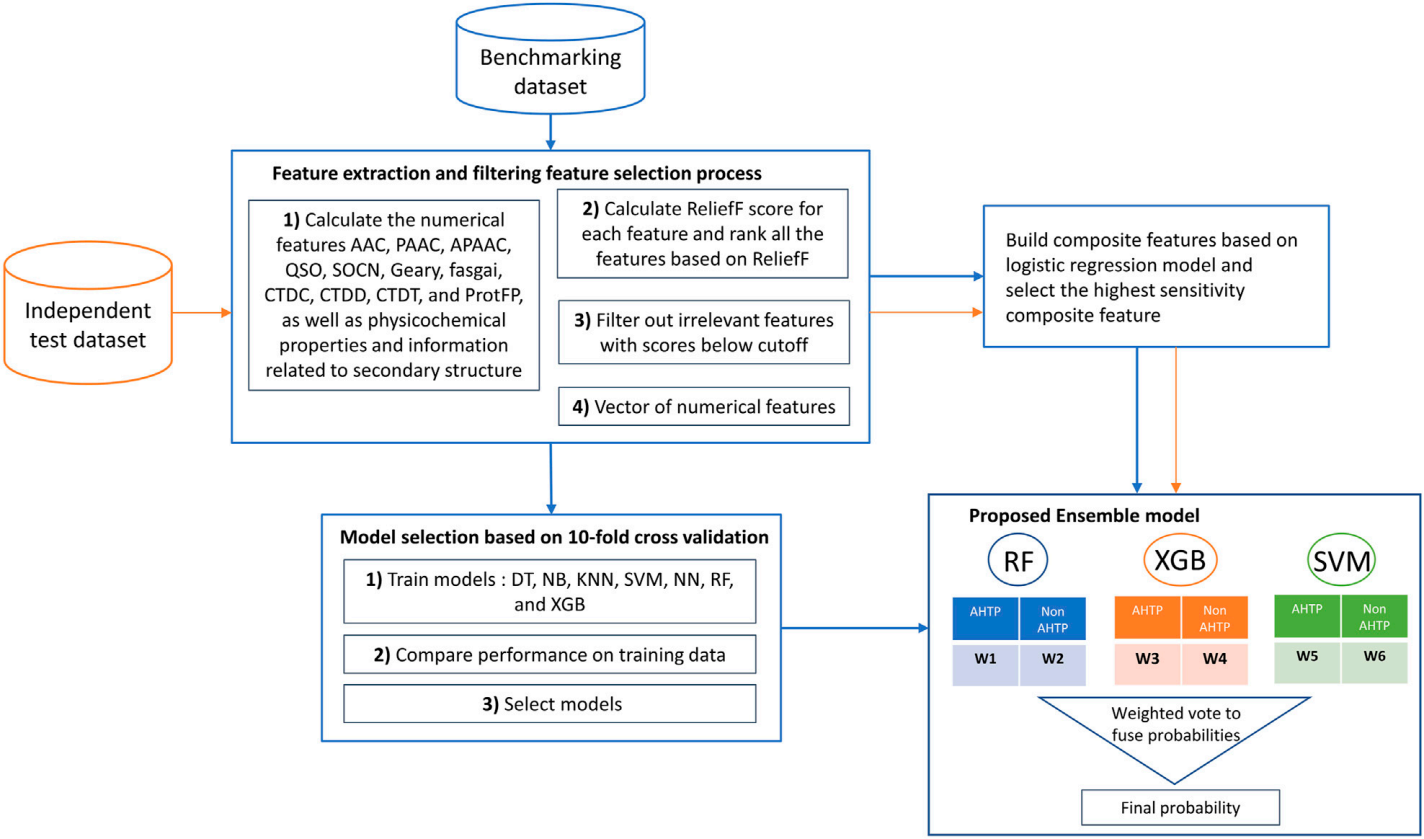
Workflow of the proposed approach.

The workflow of Ensemble-AHTPpred is shown in Figure 1.

### Datasets

In this study, we employed two nonredundant datasets from mAHTPred (Manavalan et al., 2019): a benchmarking dataset and an independent testing dataset. The balanced benchmarking dataset contained 913 unique AHTPs and 913 unique non-AHTPs. The 913 AHTPs were experimentally validated on the publicly available AHTPDB (Kumar et al., 2015) and BIOPEP (Minkiewicz et al., 2008; Iwaniak et al., 2016) databases. Note that experimentally validated non-AHTPs were not available as a public non-AHTP database. Therefore, the non-AHTPs were random peptides generated from Swiss-Prot proteins. Considering random sequences as a negative dataset is a routinely used standard procedure in many peptide-based prediction methods (Sharma et al., 2013; Kumar et al., 2015; Chen et al., 2016; Usmani et al., 2018; Manavalan et al., 2019) with the assumption that the probability of finding a random sequence to be positive is very low. Positive and negative training datasets have similar length distributions. The AHTPs in the benchmarking dataset have a length between 5 and 81 amino acids, with an average length of 7.7 amino acids. The non-AHTPs in the benchmarking dataset have a length between 5 and 45, with an average length of eight amino acids.

Another dataset, an independent dataset, was composed of 386 nonredundant, experimentally validated AHTPs (Win et al., 2018; Yi et al., 2018) and 386 random peptides generated from Swiss-Prot as negative samples. The AHTPs in the independent testing dataset have a length between 5 and 24 amino acids, with an average length of 6.48 amino acids. The non-AHTPs in the independent testing dataset have a length between 5 and 29, with an average length of 15.42 amino acids.

### Features

The peptide properties that were relevant for predicting AHTPs were determined and encoded as a vector of 431 numerical features. The features can be grouped into seven main types as follows.

1) AAC descriptors: These descriptors were used as the fractions of each amino acid type within a protein sequence. The fractions of all 20 natural amino acids {A, C, D, E, F, G, H, I, K, L, M, N, P, Q, R, S, T, V, W, Y}, were calculated. (**AAC1-AAC20: 20 dimensions**).
2) Chou’s pseudo amino acid composition (PseAAC) was generated in various modes: Chou’s PseAAC (Chou, 2005) has been widely used to convert complicated protein sequences with various lengths to fixed-length numerical feature vectors that incorporate sequence-order information. In comparison with an AAC, a PseAAC is more informative and capable of representing a protein sequence and incorporating information about its sequence order. Hence, it has been widely used for diverse amino acid sequence-based prediction problems (Chou, 2011). The PseAACs were calculated by using parameters of lambda = 3 and weight = 0.05 (**PAAC1-PAAC23: 23 dimensions**). PseAACs in parallel correlations (**Pse_PC1-Pse_PC22: 22 dimensions**), PseAACs in series correlations (**Pse_SC1-Pse_SC26: 26 dimensions**), and amphiphilic pseudo AACs with hydrophobicity correlation functions (**APAAC1_1-APAAC1_23: 23 dimensions**) and hydrophilicity correlation functions (**APAAC2_1-APAAC2_23: 23 dimensions**) were also calculated.
3) Composition/transition/distribution (C/T/D): The three descriptors based on the grouped AACs (Dubchak et al., 1995) [composition (**CTDC1-CTDC21: 21 dimensions**), transition (**CTDT1-CTDT21: 21 dimensions**) and distribution (**CTDD1-CTDD105: 105 dimensions**) descriptors] were calculated. C/T/D was calculated using the protr R package (Xiao et al., 2015). All amino acid residues were divided into three groups according to seven types of physicochemical properties, as defined in Dubchak et al. (1999). The seven physicochemical properties used for calculating these features were hydrophobicity, normalized van der Waals volume, polarity, polarizability, charge, secondary structures, and solvent accessibility.
4) Quasi-sequence-order descriptors: The quasi-sequence-order descriptors were derived from the distance matrix of the 20 amino acids (Chou 2000). Quasi-sequence-order descriptors (**QSO1-QSO46: 46 dimensions**) and sequence-order-coupling numbers (**SOCN1-SOCN6: 6 dimensions**) (lag = 3, w = 0.1) were calculated.
5) Various physicochemical and topological property-based features: The Crucian properties covariance index (**Crucian1-Crucian3: 3 dimensions**) (Cruciani et al., 2004), Z-scales based on physicochemical properties (**zscales1-zscales5: 5 dimensions**) (Sandberg et al., 1998), factor analysis scales of generalized amino acid information (**fasgai1-fasgai6: 6 dimensions**) (Liang and Li 2007), T-scales based on physicochemical properties (**tScales1-tScales5: 5 dimensions**) (Tian et al., 2007), VHSE-scales (principal component score vectors of hydrophobic, steric, and electronic properties) (**vhsescales1-vhsescales8: 8 dimensions**) (Mei et al., 2005), protFPs (**protFP1-protFP8: 8 dimensions**) (van Westen et al., 2013), ST-scales based on physicochemical properties (**stscales1-stscales8: 8 dimensions**) (Yang et al., 2010), MS-WHIM scores (**mswhimscore1-mswhimscore3: 3 dimensions**) (Zaliani and Gancia 1999), aliphatic indices of proteins (**aIndex: 1 dimension**) (Ikai, 1980), Geary autocorrelations (**geary1-geary12: 12 dimensions**), the autocovariance index (**autocov: 1 dimensions**) (Ikai, 1980), the potential protein interaction index (**Boman: 1 dimension**) (Boman, 2003), the net charge (**Charge: 1 dimension**), cross-covariance indices (**Crosscov1-Crosscov2: 2 dimensions**), instability indices (**Instaindex: 1 dimension**) (Guruprasad et al., 1990), the hmoment alpha helix (**Hmoment1: 1 dimensions**), the hmoment beta sheet (**Hmoment2: 1 dimensions**), BLOSUM matrix-derived descriptors (**Blosum1-8: 8 dimensions**), and the isoelectric point (**pI: 1 dimension**) were calculated by using the peptide R package (Osorio et al., 2015).
6) Occurrence of selected k-mer motifs: The YP, HLP, IYP, LHL, LPP, LRP, VPP, PEV, PFP, QTP, VLP, VYP, and YPF motifs (**13 dimensions**) were determined. First, we generated all 2-mers (400 dimensions) and all 3-mers (8000 dimensions). Then, we searched for the k-mer that was overrepresented in the positive and underrepresented in the negative datasets by calculating the log odds ratio score of the frequency of each k-mers in the positive versus negative datasets. Next, we ranked the discriminant k-mers based on the calculated log-odds score. Finally, we retained the top 2-mer and the top 12 3-mers as selected k-mer motif features that still need to be determined (the heatmap of log odds scores of 2-mers is shown in Figure 5).
7) Secondary structure conformation-related features: The aggregation, amyloid, turn, alpha-helix, helical aggregation, and beta-strand conformation secondary structure propensities were calculated using the Tango program (**tango1-tango6: 6 dimensions**) (Fernandez-Escamilla et al., 2004).

To further improve the prediction process with new informative features, we proposed a composite feature generation method *via* the fusion of the various selected features by using a logistic regression model. Various composite features based on various combinations of informative selected features were built by using logistic regression based on the benchmarking data and then compared through a 10-fold cross-validation process. The detailed process of building composite features is described in the hybrid feature section of ensemble-AMPPred (Lertampaiporn et al., 2021). A combination of features was used to fit a logistic regression model, which is represented by the following equation:

Prob. (Y= AHTPs|x)=logistic(x)=(eβ0+β1 X1+β2X2+β3X3+⋯+βnXn/(1+ e β0+β1X1+β2X2+β3X3+⋯+βnXn)) 

Logit transformation (the logarithm of the odds ratio that Y is in the AHTP category) was applied to link a function with the logistic regression. The logit function is defined as

$$\text{Logit}\left(\text{x}\right)\;=\text{ log}\left(\frac{\text{P}\left(Y=AHTP|X=X\right)}{P\left(Y=nonAHTP|X=X\right)}\right)=\beta_{\text{0}}\;+\;\beta_{1}X_{1}\;+\beta_{2}X_{2}\;+\;\beta_{3}X_{3}\;+\cdots +\;\beta_{\text{n}}X_{\text{n}}$$

Therefore, the composite feature was defined by the following equation:

Composite feature = β0 + β1 feature_1 +β2feature_2  + β3feature_3 +…+ βn feature_n 

where β0 is the intercept; β 1, β 2, β3 , and βn represent the regression coefficients for each selected feature in the equation; and feature_1, feature_2, …, and feature_n are the component features in the composite feature.

### Feature Selection

A feature selection procedure based on ReliefF (Kononenko, 1994) scores was used as a preprocessing step to filter irrelevant features with a cutoff score. The ReliefF score for a feature was calculated based on how well the feature could distinguish between instances that were near each other. The ReliefF evaluation criterion selected features that aided in the separation of the samples from different classes and gave higher weights to the features that discriminated the samples from the neighborhoods of different classes.

Recursive feature elimination (RFE) (Tolosi and Lengauer, 2011) is a wrapper-type feature selection algorithm. RFE starts with all features in the training dataset and then searches for a subset of features by removing features through recursive elimination to eliminate the least relevant features one by one and refitting the model. This process is repeated until the optimal number of features is reached, ensuring that the classifier can achieve high performance.

### Models

To select base classifiers for constructing an ensemble, seven machine learning algorithms were considered in our algorithm selection experiment—a naïve Bayes (NB) model, a neural network (NN), a support vector machine (SVM), k-nearest neighbors (kNN), a decision tree (DT), a random forest (RF) and an extreme gradient boost (XGB). Each algorithm has a different inductive bias and different learning hypotheses that can provide a potentially more independent and diverse set of predictions through the ensemble. For the hyperparameters, we used a grid search to find the optimal parameters.

The NB classifier is a simple probabilistic classifier based on Bayes’ theorem and substantial independence assumptions between the features.

The NN was a multilayer perceptron (MLP). An MLP is a neural network with at least three layers: an input layer, a hidden layer, and an output layer (parameters: number of epochs: 500; learning rate: 0.3; and momentum for updating weights: 0.2).

The SVM model is a supervised learning model with associated learning algorithms for data classification and regression analysis. The SVM assigns training examples to coordinates in a high-dimensional space to widen the distance between the two classes and separates the two classes with a simple hyperplane (parameters: C = 36.0; kernel = ‘Radial Basis Function’; and gamma = 0.119).

The KNN method is a well-known nonparametric technique used in statistical pattern classification due to its simplicity, intuitiveness, and effectiveness. The essential principle is that an unclassified object is assigned to the class to which the majority of its k nearest neighbors belong (parameters: k = 7 and distance = inverse weight).

The DT is another nonparametric supervised learning method used for classification and regression. It develops a model that accurately predicts the value of a target variable by inferring basic decision rules from data attributes. A tree can be thought of as an approximation to a piecewise constant (parameter: confidence factor = 0.25).

The RF algorithm is one of the most commonly used bagging ensemble algorithms because of its flexibility and ease of use. This algorithm can produce good results without hyperparameter tuning. The RF approach is an ensemble technique with the ability to achieve high accuracy and prevent overfitting by making use of voting with multiple decision trees (parameters: no. estimators = 350 and max_depth = 12).

The XGB algorithm is a gradient boosting ensemble algorithm. The boosting algorithm adjusts the model weights according to a differential loss function and then uses the adjusted weights in the next training iteration [parameters: no. estimators (nrounds) = 800; max_depth = 10; eta = 0.01; and subsample = 0.8].

The proposed method was implemented by using Perl, Python, and R scripts. The program was run on a Fedora Linux-based machine. All the data, the trained models and the standalone program are available to download at http://ncrna-pred.com/Ensemble_AHTPpred.htm.

We adopted 10-fold cross-validation to investigate the classification performance of the various models on the benchmarking dataset. Based on the 10-fold cross validation results, model selection processes were performed. Then, the best-performing models were selected based on their diverse measurements and later used as the base classifiers of the ensemble model. Thereafter, the individual base classifiers were iteratively trained to find the optimal weight for each class of each classifier. The probability weight set (w_1_, w_2_, w_3_, w_4_, w_5_, w_6_) was estimated by using the level of confidence in predicting each class (AHTP or non-AHTP), which fluctuated among the classes. The probabilities acquired from the base classifiers were aggregated through weighted voting to obtain the final prediction of the ensemble model.

Probability-weighted voting = (W_1_^∗^Prob. (RF _class=AHTP_)) + (W_2_^∗^Prob. (RF _class=non-AHTP_)) + (W_3_^∗^Prob. (XGB class = _AHTP_)) + (W_4_^∗^Prob. (XGB _class=non-AHTP_)) + (W_5_^∗^Prob (SVM _class=AHTP_)) + (W_6_^∗^Prob (SVM _class=non-AHTP_)).

To evaluate the classification performance of the model, the following metrics were used:

$$\text{ACC}=\frac{TP+TN}{\left(TP+TN+FP+FN\right)}$$

$$\text{Sn}=\frac{TP}{\left(TP+FN\right)}$$

$$\text{Sp}=\frac{TN}{\left(TN+FP\right)}$$

$$\text{MCC}=\frac{TP\times TN-FP\times FN}{\sqrt{\left(TP+FP\right)\times \left(TP+FN\right)\times \left(TN+FP\right)\times \left(TN+FN\right)}}$$

where ACC, Sn, Sp, and MCC are accuracy, sensitivity, specificity, and Matthew’s coefficient correlation, respectively. These measurements were calculated based on the numbers of true positives (TPs), true negatives (TNs), false positives (FPs) and false negatives (FNs). The area under the receiver operating characteristic (ROC) curve (AUC) was calculated to assess the tradeoff between the sensitivity and specificity performance of the different methods. The ROC curve is a plot of the TP vs. FP rates at different thresholds. For a perfect predictor, the AUC is equal to 1.

## Results and Discussion

### Amino Acid Composition and Positional Residue Analysis

The activity of peptides depends on their structure and amino acid composition. To understand the relation between the composition and antihypertensive function of a peptide, the composition of AHTPs and non-AHTPs were analyzed/investigated. Generally, most antihypertensive peptides are relatively short peptide residues with lengths that vary from 2 amino acids to 20 amino acids. The amino acid composition is a quantitative measure of the fraction of each amino acid type within a protein. The percent amino acid composition based on the physicochemical properties of amino acids (whole peptides) was computed and calculated using COPid (Kumar et al., 2008) and includes the composition of charged (DEKHR), aliphatic (ILV), aromatic (FHWY), polar (DERKQN), neutral (AGHPSTY), hydrophobic (CVLIMFW), positively charged (HKR), negatively charged (DE), tiny (ACDGST), small (EHILKMNPQV) and large (FRWY) residues, as summarized in Table 1 (a category with higher composition is shown in bold). When comparing positive and negative of benchmarking datasets, we can see that AHTPs include more aliphatic (ILV), aromatic (FHWY), and neutral (AGHPSTY) amino acid residues than non-AHTP sequences.

**TABLE 1:** Physicochemical property-based composition of amino acids.

Physicochemical property-based composition of amino acids	Positive dataset (AHTPs)	Negative dataset (non-AHTPs)
Molecular weight of the peptide (Da)	888.2	**912.5**
Number of amino acids in the sequence	7.75	**8.05**
% Composition of charged residues (DEKHR)	19.91	**24.94**
% Composition of aliphatic residues (ILV)	**22.34**	22.04
% Composition of aromatic residues (FHWY)	**14.42**	10.32
% Composition of polar residues (DERKQN)	25.81	**31.49**
% Composition of neutral residues (AGHPSTY)	**43.44**	37.68
% Composition of hydrophobic residues (CVLIMFW)	30.75	**30.83**
% Composition of positively charged residues (HKR)	12.75	**12.96**
% Composition of negatively charged residues (DE)	7.16	**11.98**
% Composition of tiny residues (ACDGST)	22.65	**34.97**
% Composition of small residues (EHILKMNPQV)	**61.94**	51
% Composition of large residues (FRWY)	**15.41**	14.03

The higher values, between the two datasets, are shown in bold.

**FIGURE 2:**
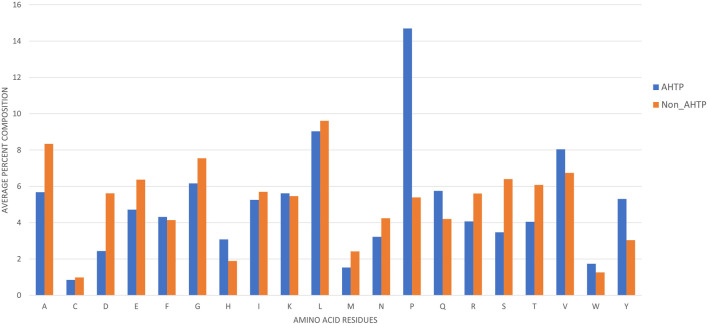
Percent average composition of amino acid residues present in the positive and negative datasets.

Amino acid residues present in AHTPs and non-AHTPs were compared, as shown in Figure 2. Histidine (H), proline (P), glutamine (Q), valine (V), tryptophan (W) and tyrosine (Y) more frequently occurred in AHTPs than in non-AHTPs, especially proline (P), which is highly abundant in AHTPs. In contrast, certain residues such as cysteine (C), aspartic acid (D), methionine (M), and tryptophan (W) occurred rarely in AHTPs. Certain types of residues occured frequently in both AHTPs and non-AHTPs, such as leucine (L) and valine (V). Amino acids such as alanine (A), aspartic Acid (D), and serine (S) were less frequent in AHTPs than in non-AHTPs.

**FIGURE 3:**
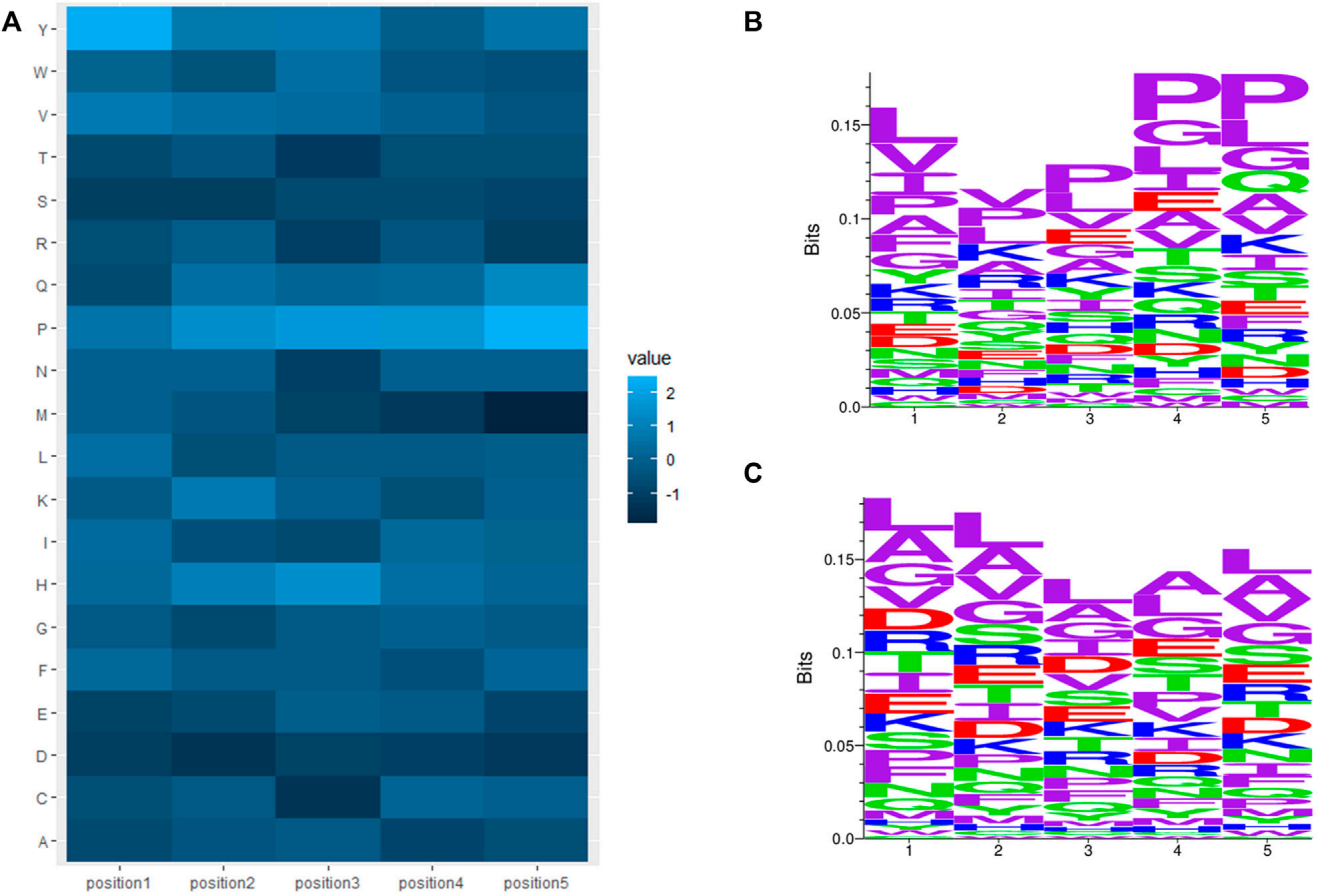
N-terminal features of AHTP positive data and non-AHTP negative data: **(A)** Heatmap of log odds ratios, where a lighter color denotes overrepresented amino acid residues in AHTPs compared to non-AHTPs (positive log odds score) and, a darker color denotes underrepresented amino acid residues in AHTPs compared to non-AHTPs (negative log odds score). **(B)** Sequence logos of positions one to five of the AHTP positive dataset. **(C)** Sequence logos of positions one to five of the non-AHTP negative dataset.

**FIGURE 4:**
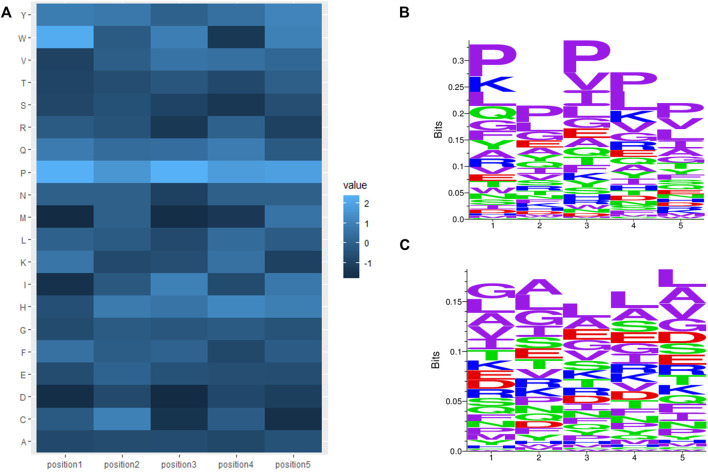
C-terminal analysis of AHTP positive data and non-AHTP negative data: **(A)** Heatmap of log odds ratios, where a lighter color denotes overrepresented amino acid residues in AHTPs compared to non-AHTPs (positive log odds score) and a darker color denotes underrepresented amino acid residues in AHTPs compared to non-AHTPs (negative log odds score). **(B)** Sequence logos of positions one to five of the AHTP positive dataset. **(C)** Sequence logos of C-terminal positions one to five of the non-AHTP negative dataset.

C-terminal and N-terminal positional residue analysis was also performed by calculating the average amino acid composition of position one to position five of the N- and C-termini in AHTPs (positive) and non-AHTPs (negative). The log odds ratios between positive and negative N- and C-termini were calculated. The log-odds ratios of positive versus negative termini were calculated as [log2 (P_a_/N_a_)], where P_a_ and N_a_ are the observed frequencies of amino acid a in the positive and negative training datasets, respectively. Heatmaps of log odds ratios were plotted for the N-terminal and C-terminal regions, as shown in Figures 3A, 4A. The sequence logos of positions one to five of the N- or C-terminus were generated by using Seq2Logo (Thomsen and Nielsen, 2012). Figures 3B,C display N-terminal positional sequence logos of AHTPs and non-AHTPs, respectively. (In sequence logos, specific colors were assigned to amino acids as follows, purple represents nonpolar sidechains (G A V L I M F W P), blue represents basic amino acid (K R H), Red represents acidic amino acid (D E), and green represents polar sidechains (S T C Y N Q); the height of the amino acids is proportional to their frequency at that position.) The most abundant amino acids in the N-terminus of AHTPs were Leu (9.069%), Pro (14.896%), Tyr (5.214%) and Val (8.697%). The most abundant amino acids in the C-terminus of AHTPs were Leu (9.003%), Pro (16.605%), and Val (7.338%). The most abundant amino acids in the N- and C-termini of non-AHTPs were Leu, Ala, Gly, and Val. The most abundant 2-mers in the N-terminus of AHTPs were YP, LP, PF, PP, and VP, while the most abundant 2-mers in the C-terminus of AHTPs were IP, FP, PL, PP, PV, QP and VP. The most abundant 2-mers in the N- and C-termini of non-AHTPs were AA, LA, AL, LG, LE, and AR.

**FIGURE 5:**
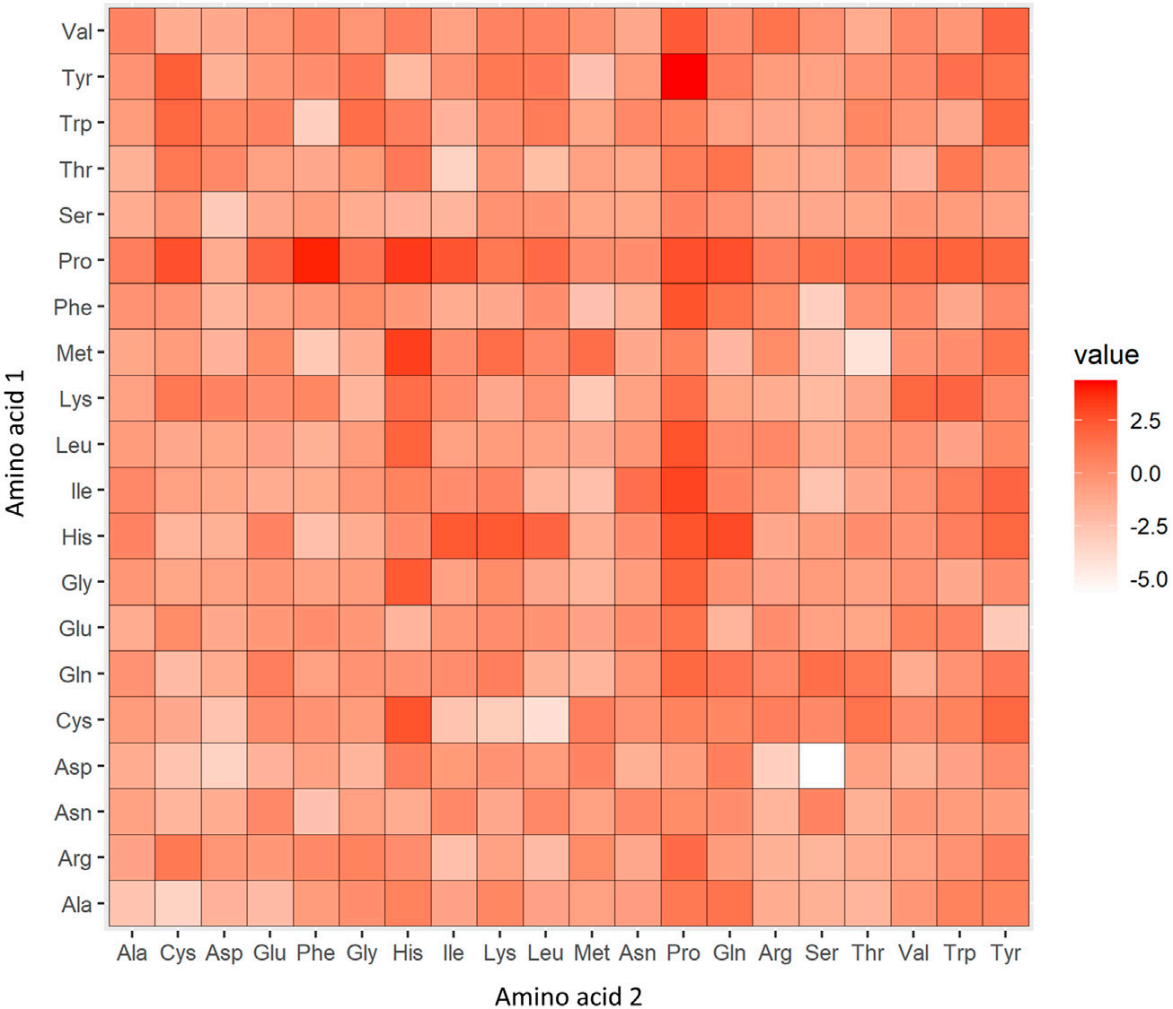
Heatmap of the log odds scores of 2-mers abundant in the positive versus negative datasets. In the heatmap, a red color (high log odds score) denotes 2-mers overrepresented in AHTPs compared to non-AHTPs, and a white color (low log odds score) denotes 2-mers underrepresented in AHTPs compared to non-AHTPs.

In addition, a heatmap of the log odds score of occurrences of the 2-mer motif in the whole sequence of AHTPs vs. in the whole sequence of non-AHTPs was also plotted, as shown in Figure 5. TyrPro (log odds = 4.393), ProPhe (log odds = 3.896) and ProHis (log odds = 3.340) were overrepresented in AHTP positive data compared to non-AHTP negative data. In contrast, AspSer (log odds = −5.708), MetThr (log odds = −4.292) and CysLeu (log odds = −4.070) were overrepresented 2-mers in non-AHTP negative data relative to AHTP positive data.

### Performance Evaluation Based on the Benchmarking Dataset to Select the Base Models for the Ensemble

Before training a prediction model, feature extraction and feature selection are two important steps for extracting various numerical features to represent biological sequences and then selecting relevant and discriminative features so that a machine learning model can further analyze and detect the generalized pattern of the data of interest. In this work, we extracted a total of 431 numerical features to represent peptide sequences.

Since we collected as many features that could explain the peptides as possible, these 431 extracted features may have contained irrelevant and noninformative features with respect to explaining the AHTPs. Feature selection is required to eliminate irrelevant and redundant features that do not explain the target class. Furthermore, feature selection mitigates the curse of dimensionality (by reducing the number of dimensions) and prevents overfitting. Filter, wrapper, and embedding techniques are the three primary feature selection methods. Both the wrapper and embedding methods are tightly coupled with specific classification algorithms. The wrapper requires one predetermined classification algorithm and relies on its performance to evaluate and select the feature subset. This approach seeks the features that are best suited to the predetermined algorithm. As a result, these methods first necessitated determining the classification algorithm to be used. However, we intended to create an ensemble consisting of multiple classification algorithms. Therefore, the filtering procedure was used initially to remove irrelevant features during this step. Note that the filtering method may not eliminate redundant features. We applied the filtering method based on ReliefF scores. After applying the filtering method, a total of 379 features had scores that were higher than the cutoff score. The vector containing these 379 numerical features was then used to train the 7 algorithms.

**FIGURE 6:**
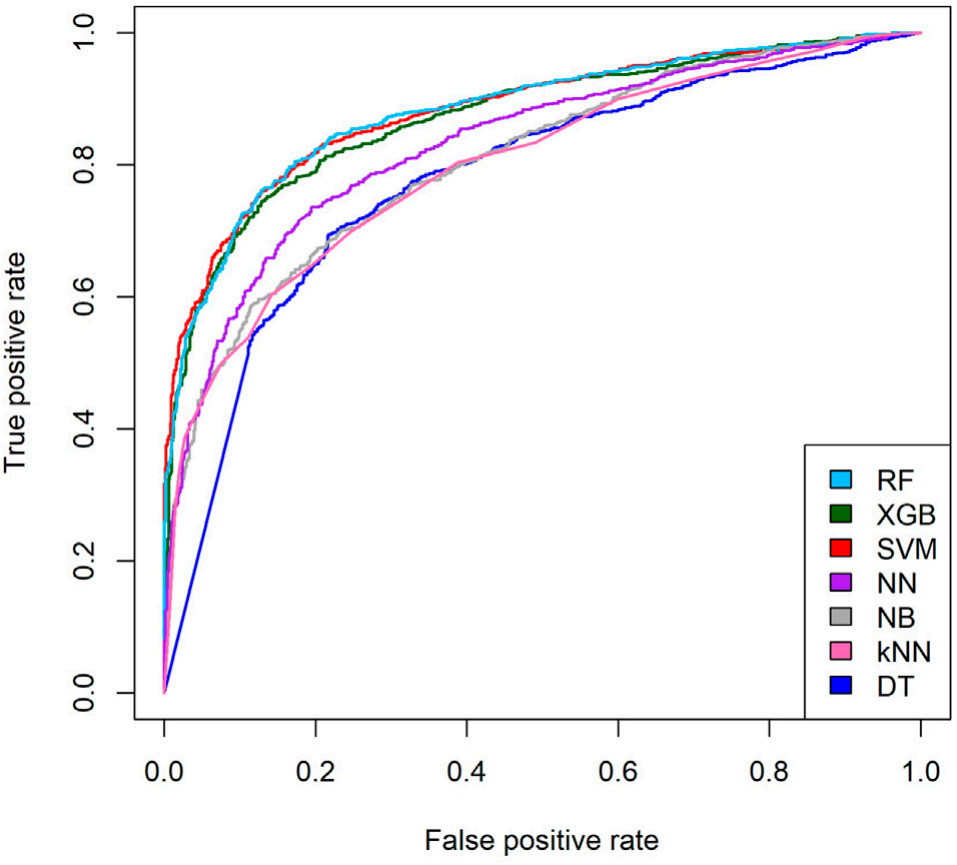
ROC curves of individual machine learning models.

The training process was carried out *via* 10-fold cross-validation on a benchmarking dataset to investigate the classification performance of different trained models. Table 2 shows the performance of the individual trained models. Different algorithms were able to take advantage of different characteristics and relationships contained in a given dataset. In this process, we detected and combined the strengths of distinct algorithms to form a resilient and stable ensemble. The findings support the “no free lunch” theorem, which states that there is no single best algorithm that is superior in terms of every metric. The ROC curves of individual classification model performance are plotted in Figure 6.

**TABLE 2:** Classification performance of different trained models.

	DT	NB	KNN	NN	SVM	XGB	RF
ACC (%)	73.494%	74.465%	74.918%	76.177%	80.504%	78.925%	**80.668%**
Sn	0.714	0.696	0.690	0.721	0.758	**0.789**	0.752
Sp	0.756	0.814	0.808	0.803	0.852	0.791	**0.861**
AUC	0.766	0.793	0.791	0.831	**0.878**	0.861	0.877

The highest values are in bold.

Based on the performance obtained during the training process, Table 2 shows that XGB had the highest sensitivity (0.789), followed by the SVM (0.758). The AUC provides a measure for evaluating which models are better on average by weighing the tradeoff between sensitivity and specificity. For the AUC metric, the SVM model achieved the highest score of 0.878, followed by the RF model (0.877), indicating that these two models achieved a good balance between positive and negative prediction. The RF model had the highest classification accuracy of 80.668% among the seven trained models. Accordingly, based on the evaluation, we chose the SVM, the RF, and XGB as the ensemble members because of their superior performance in terms of different metrics.

Note that the input vectors for the SVM model were drawn from a separate collection of features. Because the RF and XGB have built-in feature selection, we used the complete 379-feature vector as the input feed. However, for the SVM-based model, we used RFE as an additional wrapper feature selection step to remove redundant features and reduce the computational time and memory. As a result, the feature subset used as the input vector for the SVM model was reduced from 379 to 256 attributes.

Each model was assigned a weight, which was proportional to the model classification accuracy across all classes. In addition, the capacities for classification and prediction on different classes may have been unequal. Therefore, the classifier with the highest prediction confidence was given greater weight for that class. Subsequently, the training process was conducted *via* 10-fold cross validation to find the optimal class weights for each classifier/predictor in the ensemble. Thereafter, the individual classifiers (SVM, RF, and XGB) were aggregated through weighted voting to obtain the final probability and prediction.

### The New Composite Feature is Significant for Improving the Sensitivity of the Method

We propose utilizing a logistic regression equation to create additional composite features, based on the fusion of two or more existing features. In contrast to sophisticated black-box classification models, regression is a powerful way to determine the unique relationships between a large number of features and a target class. In this work, we created a number of composite features and selected the two with the highest sensitivity, which we refer to as comF and comF2. These features were merged into the feature vector as the input of the ensemble model.

The comF feature is defined as

$$\begin{array}{l} comF=0.8634-0.157tscales4-0.154CTDC19-0.135protFP6+0.133CTDC21\\ -0.132fasgai4+0.122mswhimscore1-0.12hydrophobicity \end{array}$$

The comF2 feature is defined as

$$\begin{array}{l} comF2=0.1786+0.1522APAAC1\_15-2.2951CTDC10-0.6069CTDC19-0.0065CTDD49\\ +0.2176QSO19+0.9747fasgai4+0.3691\mathrm{Pr}otFP3+2.0823Pse\_PC13 \end{array}$$

where APAAC1_15 denotes the amphiphilic PseAAC of amino acid R (the sequence-order coupling mode was used along a protein sequence *via* a hydrophobicity correlation function; the hydrophobic properties of amino acids were taken into account) and CTDC10 denotes the percentage of a particular amino acid in the polarizability group 1 (polarization between 0 and 1.08: amino acids G, A, S, D, and T) relative to protein length. CTDC19 is the percentage of a particular amino acid in solvent access group 1 (buried: amino acids A, L, F, C, G, I, V, and W) relative to the protein length. CTDD49 is the percentage of a particular amino acid in polarization group 1 (polarization between 0 and 1.08: amino acids G, A, S, D, and T) located in 75% of the residues of the protein chain. QSO19 is the quasi-sequence order of the normalized occurrence of amino acid Y, fasgai4 is a descriptor that reflects compositional characteristics, ProtFP3 is the scales-based descriptor derived from the amino acid properties of all AA indices (protein fingerprint 3), and Pse_PC13 is the parallel correlation PseAAC of amino acid P.

Interestingly, we discovered some intriguing aspects within the comF2 composite feature. Particular component properties of comF2, such as the distant locations of certain amino acids Y, R, and P, had beneficial impacts on the equation; this is consistent with the results of many research papers demonstrating that certain residues are dominant in the C-termini or N-termini of potent AHTPs. Hydrophobic residues with aliphatic side chains at the C-terminus promoted ACE inhibitory activity (Nimalaratne et al., 2015; Asoodeh et al., 2016; Jiang et al., 2021; Wang et al., 2021). Other studies have demonstrated that the positively charged lysine and arginine amino acids (K and R) contribute to the strong potency of ACE inhibitory peptides (Wei et al., 2019; Maky and Zendo, 2021). The richness of proline (P) and its number of occurrences in a sequence positively influenced the potency of ACE inhibition (Abachi et al., 2019; Festa et al., 2020; Pavlicevic et al., 2020). The presence of a polar amino acid at the C-terminus along with hydrophobic amino acids at the N-terminus may have contributed to the activity (Ryan et al., 2011; Udenigwe et al., 2012; de Castro and Sato, 2015). Moreover, the equation was adversely affected (according to the minus sign) by component properties involving low-polarization amino acids (CTDC10 and CTDD49) and those with restricted solvent access (CTDC19; buried structure).

**FIGURE 7:**
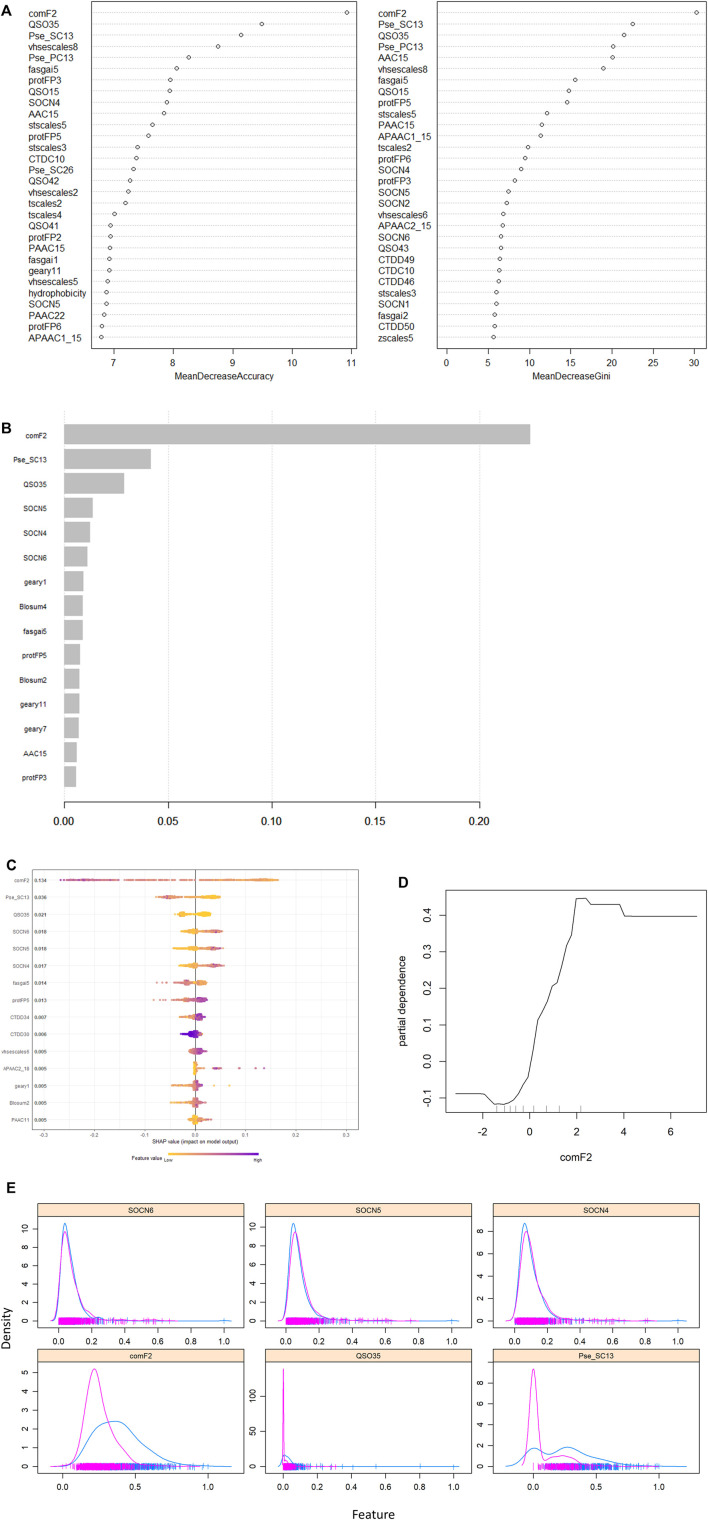
Importance plots and SHAP plot: **(A)** Importance plots yield by the RF (left: permutation importance; right: Gini importance). **(B)** Importance plot yielded by the XGB model. **(C)** SHAP summary plot of the top 15 features; **(D)** dependence plot of composite feature comF2 for the AHTP class. **(E)** Density distribution of the SHAP plot’s top six features (sample) by class in the training data.

Because the RF and XGB have built-in feature importance analysis mechanisms, we discovered that the composite feature comF2 was the highest-ranking feature in both models based on their importance plots (as shown in Figures 7A,B). It is well known that the value of a feature (as measured by information gain) varies depending on how frequently it is employed at the leaf nodes. We also conducted SHAP (Shapley Additive exPlanations) analysis as a follow-up to our initial investigation. SHAP is a game-theoretic framework for explaining the output of any machine learning model. It correlates optimal credit allocation with local explanations by using classic Shapley values (Lundberg and Lee 2017). Since it averages the marginal contributions across all permutations, the performance of SHAP is notably more consistent than that of the information gain technique. The SHAP summary plot in Figure 7C is somewhat consistent with the information gain-based importance plot, which shows that comF2 was the most significant feature, followed by Pse_SC13 and QSO35. According to the SHAP plot, the comF2 feature had an effect on the likelihoods for a larger model sample. Every dot in the SHAP plot represents a sample from the data. For each sample, the color of the corresponding dot refers to the value of the associated feature. The x-axis represents the feature’s influence on the model’s prediction. The high spread of comF2 indicates that it could capture and provide more useful information to the model to predict/identify the classes. Moreover, the partial dependence plot (PDP) of comF2 presents the impact of this feature on the predicted outcome, as shown in Figure 7D, allowing for a better understanding of the feature’s interdependence with the target class (AHTP). According to the comF2 PDP illustrated in Figure 7D, the higher the value of the comF2 feature is, the higher the chance of the sample being classified into the AHTP class by the model (comF2 greater than two likelihoods of being in the AHTP class). Additionally, Figure 7E depicts the distribution of the top six features. A substantial distribution difference was observed between the AHTP and non-AHTP classes in the histogram of the comF2 feature. However, some overlap occurred between the two classes’ territories. The functionality of comF2 can be enhanced, resulting in an increase in prediction performance.

### Comparison With Existing Prediction Methods

To evaluate the performance of the proposed method, we used the benchmarking dataset and the independence testing dataset (as shown in Tables 3, 4, respectively), and then we compared and evaluated our ensemble method with the available prediction tools based on the results reported in (Manavalan et al., 2019; Rauf et al., 2021). As shown in Table 3, our technique achieved 85.8% accuracy on the benchmarking dataset or training dataset, outperforming most of the other methods. However, while the CNN + SVM technique surpassed our ensemble for the training dataset, our ensemble performed substantially better on the independent dataset.

**TABLE 3:** Performance evaluation of the proposed method using benchmarking dataset.

Method	ACC	Sn	Sp	MCC	AUC
CNN + SVM (Rauf et al., 2021)	0.958	0.996	0.920	0.920	0.958
mAHTPred (Manavalan et al., 2019)	0.848	0.821	0.874	0.697	0.903
PAAP (Win et al., 2018)	0.791	0.865	0.780	0.585	NA
AHTpin_AAC (Kumar et al., 2015)	0.785	0.777	0.793	0.567	NA
AHTpin_ATC (Kumar et al., 2015)	0.785	0.783	0.787	0.573	NA
Our ensemble	0.858	0.832	0.885	0.718	0.926

**TABLE 4:** Performance evaluation of the proposed method using independence testing dataset.

Method	ACC	Sn	Sp	MCC	AUC
CNN + SVM (Rauf et al., 2021)	0.895	0.948	0.841	0.795	0.895
mAHTPred (Manavalan et al., 2019)	0.883	0.894	0.873	0.767	0.951
PAAP (Win et al., 2018)	NA	NA	NA	NA	NA
AHTpin_AAC (Kumar et al., 2015)	0.800	0.821	0.780	0.601	0.852
AHTpin_ATC (Kumar et al., 2015)	0.820	0.798	0.842	0.641	0.888
Our ensemble	0.904	0.920	0.889	0.809	0.965

When testing was performed on the independent data, accuracies of 90.4% were achieved, as shown in Table 4, and our method significantly outperformed the other methods.

### Performance Evaluation of Our Model With Novel Antihypertensive Peptides From Recent Studies

Novel AHTPs derived from food or natural sources are receiving significant attention. Therefore, an increasing number of food-derived or natural sources AHTPs have been researched and reported. To further assess the generalization performance and robustness of the proposed method on new unseen data, we collected various experimental AHTPs from recent studies. These published AHTPs have been validated by *in vitro* or *in vivo* experimental assays. The results are summarized in Table 5. Note that these peptides did not overlap with our training data. Our ensemble model correctly classified these novel AHTPs from different sources with an accuracy of 80%.

**TABLE 5:** Performance evaluation of the proposed method using recently reported novel AHTPs.

Peptide sequence	IC_50_	Source	References	Correctly identify by our method (Yes/No)
YLYELR	9.37 μM	Scorpion venom	Setayesh-Mehr et al. (2021)	Yes
AFPYYGHHLG	17.22 μM	Scorpion venom	Setayesh-Mehr et al. (2021)	Yes
LVLPGE	13.5 μM	Broccoli protein	Pei et al. (2021)	Yes
IPPAYTK	23.5 μM	Broccoli protein	Dang et al. (2019)	Yes
LVLPGELAK	184 μM	Broccoli protein	Dang et al. (2019)	Yes
TFQGPPHGIQVER	3.4 μM	Broccoli protein	Dang et al. (2019)	Yes
LIIPQH	120.1 μM	Rice wine lees	He et al. (2021)	Yes
LIPPEH	60.49 μM	Rice wine lees	He et al. (2021)	Yes
QTDEYGNPPR	210.03 μM	Black tea	Lu et al. (2021)	Yes
AGFAGDDAPR	178.91 μM	Black tea	Lu et al. (2021)	No
IDESLR	196.31 μM	Black tea	Lu et al. (2021)	No
IQDKEGIPPDQQR	121.11 μM	Black tea	Lu et al. (2021)	Yes
DAFGSFLYEYSE	*-*	Ricotta cheese	Pontonio et al. (2021)	No
RHPYFYAPELLYYANK	-	Ricotta cheese	Pontonio et al. (2021)	Yes
VERGRRITSV	6.82 μM	Walnut Glutelin-1	Wang et al. (2021)	No
FVIEPNITPA	6.36 μM	Walnut Glutelin-1	Wang et al. (2021)	Yes
LSGYGP	2.57 μM	Tilapia	Chen et al. (2020)	Yes
LVPPHA	414.88 μM	Radix Astragali	Wu et al. (2020)	Yes
SAGGYIW	0.002 μM	Wheat gluten	Zhang et al. (2020)	Yes
APATPSFW	0.875 μM	Wheat gluten	Zhang et al. (2020)	Yes
PPNNNPASPDFSSS	-	Soy protein	Daliri et al. (2019)	Yes
GPKALPII	-	Soy Protein	Daliri et al. (2019)	Yes
IIRCTGC	-	Soy protein	Daliri et al. (2019)	No
IGPGPFSR	47.22 μM	Mussel lamellidens	Ankhi et al. (2022)	Yes
FHAPWK	16.83 μM	Cassia obtusifolia seeds	Shih et al. (2019)	Yes

## Conclusion

In this work, an ensemble model with a combination of XGB, RF, and SVM machine learning algorithms integrated by weighted voting was developed to achieve improved sensitivity and reduce the false positive rate in terms of predicting AHTPs. A new composite feature for AHTPs, comF2, was proposed and incorporated to improve the sensitivity of the developed method. The components of the comF2 feature were selected by a machine learning process based solely on a single training dataset (benchmarking dataset). However, we hypothesize that this new feature can be improved and adjusted to be more sensitive by combining novel knowledge or the information contained in the structure-function relationships (structure-activity relationships) of AHTPs reported in recent studies or by experts/biologists in the field. This knowledge can be expanded by incorporating more recent information or new significant features found in the future to further improve the proposed approach.

Currently, deep learning (DL) has become very prominence because of its ability to identify patterns in large volumes of raw data (scalability) and its ability in perform automatic feature extraction from raw data (feature encoding/learning). However, DL does not have an explicit feature engineering step because it has automated feature extraction. We are interested in feature engineering, extraction, and selection; therefore, we apply machine learning, including DL-related algorithms so called neural nets. We exploited various features that are more explainable in terms of biological meaning, and we tried to capture an explainable relationship in the hybrid feature that may be an advantage in AHTP design in the future. We used the ensemble method, which is well-known to ensure generalization and to reduce the problem of overfitting of individual models. For precision of classification tools, both positive and negative dataset are important for model training. Availability of experimentally validated negative datasets, particularly sequences with similar amino acid compositions to those of AHTPs, will be beneficial for further improvement. Moreover, additional negative datasets containing other classes of peptides, for example, antioxidant, antimicrobial, and anticancer peptides and neuropeptides, which have been experimentally confirmed for their activities and do not show any antihypertensive activity will be more advantageous. To make this tool more useful, implementation as a webserver will be more accessible to bioactive peptide research communities.

## Data Availability

The original contributions presented in the study are included in the article/Supplementary Material, further inquiries can be directed to the corresponding author.
